# Associations of Erythrocyte Polyunsaturated Fatty Acids with Inflammation and Quality of Life in Post-Menopausal Women with Obesity Completing a Pilot Dietary Intervention

**DOI:** 10.3390/nu11071589

**Published:** 2019-07-13

**Authors:** Kylie M. Johnson, Kellie R. Weinhold, Rebecca Andridge, Kristen Arnold, Panchita P. Chu, Tonya S. Orchard

**Affiliations:** 1 Department of Human Sciences, College of Education and Human Ecology, The Ohio State University, Columbus, OH 43210, USA; 2 Division of Biostatistics, College of Public Health, The Ohio State University, Columbus, OH 43210, USA

**Keywords:** Omega-3 polyunsaturated fatty acids, inflammation, post-menopausal women, arachidonic acid, quality of life

## Abstract

Study objectives were to determine if erythrocyte omega-3 polyunsaturated fatty acids (n-3 PUFAs) increased in women participating in a dietary intervention that reduced inflammation and body weight and examine PUFA associations with markers of inflammation and quality of life (QOL). An experimental pre-post test, single group design was used. Fifteen post-menopausal women with obesity were enrolled in a 12-week pilot intervention focusing on lowering added sugars and increasing fiber and fish rich in n-3 PUFAs. Measurements included fasting blood samples, anthropometric, lifestyle and dietary data collected at baseline, end of intervention (Week 12) and follow-up (Week 24). Primary outcomes were change in erythrocyte PUFAs and associations between erythrocyte PUFAs, QOL (Short Form 12), and inflammatory markers (interleukin-6, tumor necrosis factor-α-receptor 2, and high sensitivity C-reactive protein (CRP)). Fourteen women completed all intervention visits. Mean erythrocyte docosahexaenoic acid and arachidonic acid (AA) increased at Week 12 and Week 24 (*p* < 0.001 for both), while eicosapentaenoic acid increased at Week 24 (*p* < 0.01). After adjustment for percent weight change, week 12 QOL related to physical function was significantly associated with erythrocyte linoleic acid (*p* < 0.05) and trended toward significant association with EPA (*p* = 0.051); week 24 CRP was directly associated with erythrocyte AA (*p* < 0.05). Erythrocyte n-3 PUFAs were not associated with inflammation.

## 1. Introduction

Postmenopausal, obese women are vulnerable to chronic inflammation and associated diseases [1]. Reducing chronic inflammation is associated with better health outcomes [2]. The very long-chain n-3 polyunsaturated fatty acids (n-3 PUFAs), eicosapentaenoic acid (EPA) and docosahexaenoic acid (DHA), found in high amounts in fatty fish [3], reduce circulating inflammatory markers in some [4,5,6] but not all studies [7,8]. Mechanisms underlying effects of EPA+DHA may include the low inflammatory potential of their metabolites [9]; downregulation of pro-inflammatory cytokines, including interleukin-6 (IL-6) and tumor necrosis factor-α (TNFα), [10,11]; and production of metabolites with strong inflammation resolving properties [12].

PUFAs of the n-6 family, primarily linoleic acid (LA) and arachidonic acid (AA), also have been studied in relation to inflammation [13,14]. AA-derived eicosanoids are elevated in individuals with highly inflammatory conditions but seem to have both potent pro-inflammatory and anti-inflammatory effects [15].

PUFAs have also been found to impact quality of life (QOL). Among older women with depression, supplementation with n-3 PUFAs was associated with significant improvements in physical and mental QOL [16]. Postmenopausal women supplemented with n-3 PUFAs reported improvements in menopausal-specific QOL, but not significantly different from placebo [17]. However, not all randomized controlled trials have reported benefits of n-3 PUFAs on QOL [18].

Prior analyses of data from this intervention found that a diet low in added sugars, high in fiber, and high in n-3 PUFAs from fatty fish was associated with reduced inflammation in 14 post-menopausal women with obesity who completed a 12-week pilot study [19]. In this analysis, we examined change in erythrocyte PUFAs and association with markers of inflammation and QOL in women who completed the pilot dietary intervention. We hypothesized that participants’ erythrocyte EPA and DHA would increase and be associated with lower concentrations of inflammatory markers and better QOL at end of intervention and follow-up compared to baseline.

## 2. Materials and Methods

The Ohio State University Institutional Review Board approved this study and all participants provided written informed consent. An experimental pre-post test, single group design was used. The pilot study, evaluating a 12-week dietary intervention to reduce inflammation, is described in detail elsewhere [19]. Briefly, participants received weekly tailored nutrition counseling for 12 weeks. Specific study goals included increasing fatty fish intake to ≥3 servings/week, increasing fiber intake to ≥20 g/day, and reducing added sugar intake to ≤10% of total kcals/day. A 12-week maintenance period without contact from researchers followed the end of the intervention, and participants returned for one follow-up visit 24-weeks from baseline.

A sample size of 15 was chosen to provide >80% power to detect changes in nutritional biomarkers (e.g., plasma and red blood cell n-3 PUFAs), based on previously reported effect sizes [20]. Inclusion criteria included females age ≥40 years with obesity (BMI > 30 kg/m^2^) who were post-menopausal (defined as self-reported amenorrhea ≥12 months) and willing to make dietary changes. Exclusion criteria included self-reported current inflammatory disease, type 1 or type 2 diabetes mellitus, symptomatic heart disease, and daily use of NSAIDs, aspirin, hypoglycemic medications, or high dose n-3 PUFA supplement (>360 mg EPA + DHA/day). Volunteers were recruited from central Ohio and enrolled in 2015. An in-person visit confirmed eligibility via height and weight measurement to calculate BMI.

Outcome measurements were collected at baseline, end of intervention (week 12) and follow-up (week 24). Body weight and height were measured using a research quality scale and stadiometer (Seca Co., Hamburg, Germany). Usual dietary intake over the previous twelve weeks was assessed using Vioscreen, a validated web-based tool including a graphical self-administered electronic food frequency questionnaire (VioCare, Inc., Princeton, NJ, USA) [21]. Self-reported QOL was assessed using the 12-Item Short-Form Health Survey (SF-12), a valid and reliable tool for measuring physical and mental QOL [22].

Fasting whole blood was collected and stored at −80 °C. Serum inflammatory markers, IL-6, TNFα receptor-2 (TNFα R-2), and high sensitivity C-reactive protein (CRP), were analyzed by electrochemilluminescence and read using the Meso Quick Plex SQ 120 (Meso Scale, Discovery, Rockville, MD, USA) at the Ohio State University Clinical Research Center as previously reported [19].

Lipids were extracted from erythrocytes, and fatty acids (FAs) were methylated using boron trifluoride as previously described [23,24,25]. FA methyl esters were analyzed using a gas chromatograph (Shimadzu Scientific Instruments, Columbia, MD, USA) equipped with a 30-m Omegawax TM 320 fused silica capillary column (Supelco, Bellefonte, PA, USA) according to established methods [26]. Retention times of samples were compared to standards (Matreya, LLC, Pleasant Gap, PA, USA; Supelco, Bellefonte, PA, USA; and Nu-Check Prep Inc., Elysian, MN, USA). FAs are reported as a percent of total FAs [27]. Representative coefficients of variation from eight duplicate samples were: EPA, 6.6%; DHA, 3.8%; LA, 1.2%; AA, 0.9%.

The primary objectives of this analysis were to (1) determine the effects of a dietary intervention on erythrocyte PUFA composition; and (2) to examine the relationships between erythrocyte PUFAs and inflammatory markers. The secondary objectives were to determine correlations among self-reported intake of dietary PUFAs, QOL outcomes, and erythrocyte PUFAs. All analyses were conducted using JMP (version 11.2.1, SAS Institute Inc., Cary, NC, USA). Repeated measures analysis of variance and post-hoc t-tests evaluated changes over time in erythrocyte FAs, inflammatory markers, QOL outcomes, and dietary variables. Pearson correlations quantified cross-sectional associations between erythrocyte FAs, inflammatory markers, QOL outcomes, and dietary variables. All dietary data were energy adjusted prior to correlation analyses. Due to significant weight loss observed post-intervention and at follow-up [19], partial correlations were used to adjust for percent weight change, a potential confounder of the relationships of erythrocyte FAs with inflammatory markers and QOL outcomes.

## 3. Results

### 3.1. Self-Reported Dietary Intake

Self-reported intake of dietary PUFAs (g/day) is shown in Table 1. Intake of fatty fish, added sugars, fiber, and energy are reported elsewhere [19]. Self-reported intake of EPA and DHA increased and LA decreased significantly from baseline to intervention end; increases in DHA and EPA remained statistically higher than baseline at follow-up (all *p* < 0.017). When analyzed as a percent of total energy intake, similar trends were observed for both EPA and DHA (all *p* < 0.001), but no significant differences were observed for LA. Self-reported intake of AA did not change significantly, however AA as a percent of total energy intake increased significantly from baseline to intervention end (*p* < 0.0001).

### 3.2. Erythrocyte PUFA Content

Erythrocyte EPA and DHA content significantly increased over the course of this study (Table 2). EPA did not significantly increase until week 24 (*p* = 0.007). However, DHA, EPA+DHA (also called the Omega-3 Index), and AA increased from baseline to week 12 (*p* < 0.001 for all) and remained significantly higher than baseline at week 24 (*p* < 0.001 for all). Despite decreases in dietary LA (g/d) over the study period, erythrocyte LA did not change significantly.

### 3.3. Association of Dietary Variables and Erythrocyte PUFA Content

Dietary EPA and DHA were strongly and positively correlated with erythrocyte EPA and DHA (Table 3) at baseline, week 12 and week 24 (*p* < 0.05 for all). Similar correlations were noted between self-reported intake of fatty fish and erythrocyte DHA, as well as the Omega-3 Index at week 12 (*p* < 0.01 for both; data not shown). Dietary LA and AA were not significantly associated with erythrocyte LA and AA.

### 3.4. Association of Erythrocyte PUFAs and Inflammatory Markers

Levels of serum inflammatory markers at each time point have been previously reported [19]. Mean TNFαR-2 reduced significantly from baseline (5983 pg/mL) to post-intervention (5206 pg/mL, *p* < 0.01), and from baseline to follow-up (5012 pg/mL, *p* < 0.001). CRP levels trended downward at each visit, however changes were not significant. Erythrocyte EPA, DHA and the Omega-3 Index were not associated with TNFαR-2, CRP or IL-6 in unadjusted analyses (data not shown), or when controlling for percent weight change (Table 4). A direct relationship was observed between erythrocyte AA content and CRP at follow-up when controlling for percent weight change from week 12 to week 24 (r = 0.59, *p* = 0.04).

### 3.5. Association of Erythrocyte PUFAs and Quality of Life

Measures of QOL did not change significantly over the duration of the intervention and follow-up (Table 5). Physical function score increased slightly from baseline to end of intervention, but then reduced to slightly below baseline at follow-up (*p* > 0.05). Mental function score increased from baseline to end of intervention, and the trend continued at follow-up, however changes were not statistically significant (all *p* > 0.05). Cross-sectional associations between QOL outcomes and erythrocyte PUFA content at end of intervention and follow-up, controlling for changes in body weight, are reported in Table 6. Physical function was directly related to erythrocyte LA at end of intervention (r = 0.58, *p* = 0.037), and neared significance with erythrocyte EPA and EPA+DHA at end of intervention (r = 0.55, *p* = 0.051; r = 0.50, *p* = 0.085, respectfully). Physical function was not significantly associated with any erythrocyte PUFA measures at follow-up. Mental function was not significantly associated with any erythrocyte PUFA measures at end of intervention or follow-up.

## 4. Discussion

The results of this analysis support the efficacy of this behaviorally-based dietary intervention to raise biomarkers of n-3 PUFA intake. However, despite significant increases in erythrocyte EPA and DHA, and reductions in TNFαR-2 over the course of the study [19], erythrocyte n-3 PUFAs were not associated with any measured inflammatory marker. Higher erythrocyte AA was associated with significantly higher CRP. Higher erythrocyte LA was associated with better physical functioning on the SF-12 QOL assessment at the end of the intervention.

As women in this study increased fatty fish intake to reach the goal of three 3-oz. servings per week [19], dietary EPA and DHA increased by 0.12 g/day (0.07% kcal) and 0.24 g/day (0.14% kcal) respectively, by end of the intervention. The mean value of the Omega-3 index increased by 1.37% from baseline, and self-reported intakes of EPA and DHA were significantly associated with their respective erythrocyte PUFAs at all time points. These results suggest that participants experienced a dose response increase in erythrocyte EPA and DHA with increasing dietary EPA and DHA, as reported by others [25,28,29].

Potential explanations for the lack of association between erythrocyte EPA or DHA and TNFαR-2, IL-6 or CRP exist. Participants may not have consumed enough n-3 PUFAs to realize anti-inflammatory benefits despite meeting targeted fatty fish intake. Women in the current study achieved a mean intake of EPA+DHA of 0.52 g/day by the end of the intervention. Although within the range recommended for cardiovascular benefit [29], this amount may not be sufficient to reduce chronic inflammation [5], particularly as those with obesity may require a larger dose of n-3 PUFAs to achieve the same targeted erythrocyte level as lean counterparts [4]. Additionally, women in this study had a low baseline Omega-3 index of 3.54%, even less than the typically low Omega-3 Index of ~4–4.5% reported in adults not taking fish oil supplements and consuming a Western-style diet [29]. Participants reached a mean Omega-3 index of 4.91% by the end of the intervention but did not approach the level of 8% suggested by some researchers as optimal [30], although this target is based on cardiovascular benefit. It is also possible that our sample size was too small to detect relationships between n-3 PUFAs and inflammation, as power analysis was based on change in PUFA biomarkers as described previously [19].

Mean erythrocyte AA content significantly increased from baseline to follow-up in our participants; however, erythrocyte AA was not associated with dietary intake of AA. Increasing erythrocyte AA could be related to our participants’ weight loss. In some studies of obese adults with non-alcoholic fatty liver disease [31,32], AA in erythrocyte phospholipids was low compared to lean controls. Weight reduction following bariatric surgery resulted in normalization of erythrocyte AA in these patients [32]. Similarly, diet-induced weight reduction of >5% increased erythrocyte AA, improved erythrocyte membrane fluidity, and improved insulin sensitivity in overweight and moderately obese women [33]. The authors suggest that increasing membrane fluidity via increasing AA may contribute to some of the beneficial metabolic outcomes associated with weight loss [33]. Despite this potential metabolic benefit, we observed a direct association between erythrocyte AA and inflammation (i.e., CRP) in women at follow-up, although overall CRP trended downward in our study. Similar to our results, AA levels in blood and tissue have been associated with increased inflammation in some studies [4,15]. Reductions in erythrocyte AA have been reported when supplemental EPA+DHA was provided to healthy lean adults, but not until doses reached 0.6 g/day [14,29], which is greater than our participants’ mean intake. Taken together, these studies support the crucial role of adequate, but not excessive, AA in cellular membranes.

At the end of the intervention, higher erythrocyte LA was significantly associated with a better physical function score on the SF-12 QOL measure after adjusting for percent weight change. Similarly, higher erythrocyte EPA approached significance (*p* = 0.051) with better physical function score. Beneficial effects of EPA and DHA supplementation on physical and mental function scores from the SF-36, a more comprehensive QOL tool, have been reported in elderly, depressed women who were supplemented with 2.5 g of EPA+DHA versus paraffin placebo for two months in a randomized, double blind, controlled trial [16]. In another randomized, controlled trial of elderly people with mild cognitive impairment, approximately 2 g of either EPA, DHA or LA were supplemented for 6 months; despite improvements in depressive symptoms with EPA and DHA versus LA, none of the supplements significantly affected QOL measured by SF-36 [34]. Women in our study did not consume as high a dose of EPA and DHA as achieved in these supplementation studies, but still appeared to obtain some marginal benefit on self-reported QOL by increasing EPA and maintaining LA.

Limitations of this pilot study include the small sample size with minimal diversity and lack of a control group. Additionally, women lost weight during the intervention, which could have been a confounding factor; we addressed this by adjusting analyses for weight change. Strengths of this study include the use of a validated, objective biomarker of EPA and DHA intake and n-6 PUFA status, and measurement of multiple biomarkers of inflammation across time.

## 5. Conclusions

Erythrocyte EPA, DHA and AA were higher and inflammation was lower following a dietary intervention to reduce inflammation in post-menopausal women with obesity. However, erythrocyte EPA and DHA were not associated with inflammation at any time point. Erythrocyte EPA and LA were associated with better self-reported physical functioning at the end of the intervention. Testing this intervention in a larger sample with a more rigorous study design is warranted to determine if these changes produce beneficial health outcomes.

## Figures and Tables

**Table 1:** Self-reported intake of dietary polyunsaturated fatty acids (PUFA) in fourteen post-menopausal women completing the LASO-3 intervention.

	Baseline	End of Intervention	Change from Baseline to Intervention End	Follow-Up	Change from Baseline to Follow-Up
Dietary PUFA ^1^	Mean (SD)	Mean (SD)	*p*-Value ^2^	Mean (SD) ^3^	*p*-Value ^2^
EPA (g/day)	0.05 (0.07)	0.17 (0.13)	<0.0001	0.14 (0.13)	0.001
EPA (% kcal)	0.02 (0.03)	0.09 (0.07)	<0.0001	0.08 (0.06)	0.0006
DHA (g/day)	0.11 (0.15)	0.35 (0.27)	<0.0001	0.30 (0.26)	0.0006
DHA (% kcal)	0.05 (0.06)	0.19 (0.13)	<0.0001	0.16 (0.13)	0.0002
EPA+DHA (g/day)	0.16 (0.22)	0.52 (0.40)	<0.0001	0.45 (0.39)	0.0008
EPA+DHA (% kcal)	0.07 (0.10)	0.28 (0.20)	<0.0001	0.24 (0.19)	0.0003
LA (g/day)	15.9 (7.3)	11.8 (4.2)	0.005	12.9 (5.7)	0.046
LA (% kcal)	6.9 (1.6)	6.5 (1.8)	0.48	6.8 (1.9)	0.91
AA (g/day)	0.09 (0.06)	0.10 (0.05)	0.12	0.09 (0.04)	0.65
AA (% kcal)	0.04 (0.02)	0.06 (0.02)	<0.0001	0.05 (0.02)	0.04

^1^ Data taken from electronic Food Frequency Questionnaires, estimating intake of foods and beverages over the previous 12 weeks, presented per day; ^2^ Repeated measures analyses of variance and post-hoc student t-tests determined differences in variables between time points; ^3^ *n* = 13; one participant missing dietary data at follow-up.

**Table 2:** Erythrocyte PUFA measurement in fourteen post-menopausal women completing the LASO-3 intervention.

	Baseline	End of Intervention	Change from Baseline to Intervention End	Follow-Up	Change from Baseline to Follow-Up
Erythrocyte PUFA ^1^	Mean (SD)	Mean (SD)	*p*-Value ^2^	Mean (SD) ^3^	*p*-Value ^2^
EPA (%)	0.47 (0.27)	0.62 (0.37)	0.03	0.68 (0.36)	0.007
DHA (%)	3.1 (1.0)	4.3 (1.1)	<0.0001	4.2 (1.1)	<0.0001
EPA+DHA (%)	3.5 (1.3)	4.9 (1.5)	<0.0001	4.9 (1.4)	0.0001
LA (%)	12.5 (2.2)	12.4 (1.8)	0.79	12.6 (1.7)	0.92
AA (%)	15.0 (2.0)	16.27 (1.42)	0.0007	16.50 (1.65)	0.0002

^1^ Data reported as percent of total fatty acids measured by gas chromatography; ^2^ Repeated measures analyses of variance and post-hoc student t-tests determined differences in variables between time points; ^3^ *n* = 13; one participant missing blood work at follow-up.

**Table 3:** Associations between self-reported dietary intake and erythrocyte PUFA content among fourteen post-menopausal women completing the LASO-3 intervention.

		Baseline		End of Intervention		Follow-Up ^1^	
Dietary PUFA ^2^	Erythrocyte PUFA ^3^	r	*p*-Value ^4^	r	*p*-Value	r	*p*-Value
EPA	EPA	0.72	0.004	0.61	0.02	0.72	0.009
DHA	DHA	0.71	0.005	0.71	0.005	0.78	0.003
EPA+DHA	EPA+DHA	0.72	0.004	0.70	0.005	0.78	0.003
LA	LA	−0.22	0.44	0.16	0.59	−0.03	0.93
AA	AA	−0.18	0.53	−0.07	0.81	−0.24	0.45

^1^*n* = 12 (follow-up), two subjects excluded at follow-up due to missing data; ^2^ Total grams per day of dietary polyunsaturated fatty acid (PUFA) estimated from electronic food frequency questionnaire; ^3^ Erythrocyte polyunsaturated fatty acid (PUFA) content reported as percent of total erythrocyte fatty acids measured by gas chromatography; ^4^ Pairwise Pearson correlations assessed associations between dietary and erythrocyte PUFAs at each time point.

**Table 4:** Associations between inflammatory markers and erythrocyte PUFA, adjusting for % weight change, among fourteen post-menopausal women completing the LASO-3 intervention.

		End of Intervention		Follow-Up	
Inflammatory Marker	Erythrocyte PUFA ^1^	r ^2^	*p*-Value	r ^3,4^	*p*-Value
TNFaR-2	EPA	0.13	0.68	0.34	0.27
	DHA	0.40	0.18	0.31	0.33
	EPA+DHA	0.34	0.25	0.33	0.30
	LA	−0.41	0.16	−0.32	0.31
	AA	0.24	0.44	0.02	0.95
IL-6	EPA	−0.24	0.43	0.22	0.49
	DHA	−0.10	0.75	0.24	0.46
	EPA+DHA	−0.13	0.66	0.24	0.46
	LA	0.03	0.93	−0.52	0.08
	AA	0.29	0.34	0.20	0.54
CRP **^5^**	EPA	−0.43	0.15	−0.30	0.35
	DHA	−0.42	0.15	−0.33	0.29
	EPA+DHA	−0.43	0.30	−0.33	0.48
	LA	0.04	0.89	0.00	1.00
	AA	−0.11	0.72	0.59	0.04

^1^ Erythrocyte polyunsaturated fatty acid (PUFA) content reported as percent of total erythrocyte fatty acids measured by gas chromatography; ^2^ Partial correlation adjusting for percent weight change from week 1 to week 12; ^3^ Partial correlation adjusting for percent weight change from week 12 to week 24; ^4^ *n* = 13, one participant missing blood data at follow-up; ^5^ Variable was natural log transformed to meet model assumptions.

**Table 5:** Quality of Life measurement in fourteen post-menopausal women completing the LASO-3 intervention.

	Baseline	End of Intervention	Change from Baseline to Intervention End	Follow-Up	Change from Baseline to Follow-Up
Quality of Life Outcome ^1^	Mean (SD)	Mean (SD)	*p*-Value ^2^	Mean (SD)	*p*-Value ^2^
Physical function score	51.26 (6.59)	52.72 (5.98)	0.59	49.41 (8.49)	0.49
Mental function score	47.93 (12.33)	50.70 (6.93)	0.46	52.39 (9.74)	0.24

^1^ Summary scores for physical and mental health function from SF-12 questionnaire. Scores range from 0 (lowest level) to 100 (highest level); ^2^ Repeated measures analyses of variance and post-hoc student t-tests determined differences in variables between time points.

**Table 6:** Associations between quality of life indicators and erythrocyte polyunsaturated fatty acids (PUFA), adjusting for % weight change, among fourteen post-menopausal women completing the LASO-3 intervention.

		End of Intervention		Follow-Up	
Quality of Life Outcome ^1^	Erythrocyte PUFA ^2^	r ^3^	*p*-Value	r ^4^	*p*-Value
Physical function score	EPA	0.55	0.05	0.09	0.76
	DHA	0.46	0.11	0.24	0.45
	EPA+DHA	0.50	0.08	0.20	0.51
	LA	0.58	0.04	0.03	0.93
	AA	−0.34	0.26	−0.02	0.94
Mental function score	EPA	0.21	0.49	0.19	0.60
	DHA	0.23	0.46	0.08	0.83
	EPA+DHA	0.23	0.45	0.11	0.77
	LA	−0.28	0.34	0.03	0.93
	AA	0.03	0.91	−0.23	0.53

^1^ Summary scores for physical and mental health function from SF-12 questionnaire; ^2^ Erythrocyte polyunsaturated fatty acid (PUFA) content reported as percent of total erythrocyte fatty acids measured by gas chromatography; ^3^ Partial correlation adjusting for percent weight change from week 1 to week 12; ^4^ Partial correlation adjusting for percent weight change from week 12 to week 24.

## References

[1] Nakamura K., Fuster J.J., Walsh K. (2014). Adipokines: A link between obesity and cardiovascular disease. J. Cardiol..

[2] Heydari B., Abdullah S., Pottala J.V., Shah R., Abbasi S., Mandry D., Francis S.A., Lumish H., Ghoshhajra B.B., Hoffmann U. (2016). Effect of Omega-3 Acid Ethyl Esters on Left Ventricular Remodeling After Acute Myocardial Infarction: The OMEGA-REMODEL Randomized Clinical Trial. Circulation.

[3] Gebauer S.K., Psota T.L., Harris W.S., Kris-Etherton P.M. (2006). n-3 fatty acid dietary recommendations and food sources to achieve essentiality and cardiovascular benefits. Am. J. Clin. Nutr..

[4] Flock M.R., Skulas-Ray A.C., Harris W.S., Gaugler T.L., Fleming J.A., Kris-Etherton P.M. (2014). Effects of supplemental long-chain omega-3 fatty acids and erythrocyte membrane fatty acid content on circulating inflammatory markers in a randomized controlled trial of healthy adults. Prostaglandins Leukot. Essent. Fat. Acids.

[5] Lopez-Garcia E., Schulze M.B., Manson J.E., Meigs J.B., Albert C.M., Rifai N., Willett W.C., Hu F.B. (2004). Consumption of (n-3) fatty acids is related to plasma biomarkers of inflammation and endothelial activation in women. J. Nutr..

[6] He K., Liu K., Daviglus M.L., Jenny N.S., Mayer-Davis E., Jiang R., Steffen L., Siscovick D., Tsai M., Herrington D. (2009). Associations of dietary long-chain n-3 polyunsaturated fatty acids and fish with biomarkers of inflammation and endothelial activation (from the Multi-Ethnic Study of Atherosclerosis [MESA]). Am. J. Cardiol..

[7] Poreba M., Mostowik M., Siniarski A., Golebiowska-Wiatrak R., Malinowski K.P., Haberka M., Konduracka E., Nessler J., Undas A., Gajos G. (2017). Treatment with high-dose n-3 PUFAs has no effect on platelet function, coagulation, metabolic status or inflammation in patients with atherosclerosis and type 2 diabetes. Cardiovasc. Diabetol..

[8] Hames K.C., Morgan-Bathke M., Harteneck D.A., Zhou L., Port J.D., Lanza I.R., Jensen M.D. (2017). Very-long-chain ω-3 fatty acid supplements and adipose tissue functions: a randomized controlled trial. Am. J. Clin. Nutr..

[9] Von Schacky C., Fischer S., Weber P.C. (1985). Long-term effects of dietary marine omega-3 fatty acids upon plasma and cellular lipids, platelet function, and eicosanoid formation in humans. J. Clin. Investig..

[10] Novak T.E., Babcock T.A., Jho D.H., Helton W.S., Espat N.J. (2003). NF-kappa B inhibition by omega -3 fatty acids modulates LPS-stimulated macrophage TNF-alpha transcription. Am. J. Physiol. Lung. Cell. Mol. Physiol..

[11] Calder P.C., Grimble R.F. (2002). Polyunsaturated fatty acids, inflammation and immunity. Eur. J. Clin. Nutr..

[12] Serhan C.N., Clish C.B., Brannon J., Colgan S.P., Chiang N., Gronert K. (2000). Novel functional sets of lipid-derived mediators with antiinflammatory actions generated from omega-3 fatty acids via cyclooxygenase 2-nonsteroidal antiinflammatory drugs and transcellular processing. J. Exp. Med..

[13] Novgorodtseva T.P., Denisenko Y.K., Zhukova N.V., Antonyuk M.V., Knyshova V.V., Gvozdenko T.A. (2013). Modification of the fatty acid composition of the erythrocyte membrane in patients with chronic respiratory diseases. Lipids Health Dis..

[14] Wood K.E., Lau A., Mantzioris E., Gibson R.A., Ramsden C.E., Muhlhausler B.S. (2014). A low omega-6 polyunsaturated fatty acid (n-6 PUFA) diet increases omega-3 (n-3) long chain PUFA status in plasma phospholipids in humans. Prostaglandins Leukot. Essent Fat. Acids.

[15] Innes J.K., Calder P.C. (2018). Omega-6 fatty acids and inflammation. Prostaglandins Leukot. Essent Fat. Acids.

[16] Rondanelli M., Giacosa A., Opizzi A., Pelucchi C., La Vecchia C., Montorfano G., Negroni M., Berra B., Politi F., Rizzo A.M. (2011). Long chain omega 3 polyunsaturated fatty acids supplementation in the treatment of elderly depression: effects on depressive symptoms, on phospholipids fatty acids profile and on health-related quality of life. J. Nutr. Health Aging.

[17] Lucas M., Asselin G., Merette C., Poulin M.J., Dodin S. (2009). Effects of ethyl-eicosapentaenoic acid omega-3 fatty acid supplementation on hot flashes and quality of life among middle-aged women: a double-blind, placebo-controlled, randomized clinical trial. Menopause.

[18] Andreeva V.A., Latarche C., Hercberg S., Briançon S., Galan P., Kesse-Guyot E. (2014). B vitamin and/or n-3 fatty acid supplementation and health-related quality of life: ancillary findings from the SU.FOL.OM3 randomized trial. PLoS ONE.

[19] Arnold K., Weinhold K.R., Andridge R., Johnson K., Orchard T.S. (2018). Improving Diet Quality Is Associated with Decreased Inflammation: Findings from a Pilot Intervention in Postmenopausal Women with Obesity. J. Acads. Nutr. Diet..

[20] Harris W.S., Pottala J.V., Sands S.A., Jones P.G. (2007). Comparison of the effects of fish and fish-oil capsules on the n 3 fatty acid content of blood cells and plasma phospholipids. Am. J. Clin. Nutr..

[21] Kristal A.R., Kolar A.S., Fisher J.L., Plascak J.J., Stumbo P.J., Weiss R., Paskett E.D. (2014). Evaluation of web-based, self-administered, graphical food frequency questionnaire. J. Acads. Nutr. Diet..

[22] Ware J., Kosinski M., Keller S.D. (1996). A 12-Item Short-Form Health Survey: construction of scales and preliminary tests of reliability and validity. Med. Care.

[23] Pottala J.V., Espeland M.A., Polreis J., Robinson J., Harris W.S. (2012). Correcting the effects of -20 degrees C storage and aliquot size on erythrocyte fatty acid content in the Women’s Health Initiative. Lipids.

[24] Morrison W.R., Smith L.M. (1964). Preparation of fatty acid methyl esters and dimethylacetals from lipids with boron fluoride--methanol. J. Lipid Res..

[25] Harris W.S., Lemke S.L., Hansen S.N., Goldstein D.A., DiRienzo M.A., Su H., Nemeth M.A., Taylor M.L., Ahmed G., George C. (2008). Stearidonic acid-enriched soybean oil increased the omega-3 index, an emerging cardiovascular risk marker. Lipids.

[26] Belury M.A., Kempa-Steczko A. (1997). Conjugated linoleic acid modulates hepatic lipid composition in mice. Lipids.

[27] Orchard T.S., Ing S.W., Lu B., Belury M.A., Johnson K., Wactawski-Wende J., Jackson R.D. (2013). The association of red blood cell n-3 and n-6 fatty acids with bone mineral density and hip fracture risk in the women’s health initiative. J. Bone Miner. Res..

[28] Straka S., Lester J.L., Cole R.M., Andridge R.R., Puchala S., Rose A.M., Clinton S.K., Belury M.A., Yee L.D. (2015). Incorporation of eicosapentaenioic and docosahexaenoic acids into breast adipose tissue of women at high risk of breast cancer: a randomized clinical trial of dietary fish and n-3 fatty acid capsules. Mol. Nutr. Food Res..

[29] Flock M.R., Skulas-Ray A.C., Harris W.S., Etherton T.D., Fleming J.A., Kris-Etherton P.M. (2013). Determinants of erythrocyte omega-3 fatty acid content in response to fish oil supplementation: a dose-response randomized controlled trial. J. Am. Heart Assoc..

[30] Harris W.S. (2007). Omega-3 fatty acids and cardiovascular disease: A case for omega-3 index as a new risk factor. Pharmacol. Res..

[31] Elizondo A., Araya J., Rodrigo R., Poniachik J., Csendes A., Maluenda F., Diaz J.C., Signorini C., Sgherri C., Comporti M. (2007). Polyunsaturated fatty acid pattern in liver and erythrocyte phospholipids from obese patients. Obesity (Silver Spring).

[32] Elizondo A., Araya J., Rodrigo R., Signorini C., Sgherri C., Comporti M., Poniachik J., Videla L.A. (2008). Effects of weight loss on liver and erythrocyte polyunsaturated fatty acid pattern and oxidative stress status in obese patients with non-alcoholic fatty liver disease. Biol. Res..

[33] Cazzola R., Rondanelli M., Trotti R., Cestaro B. (2011). Effects of weight loss on erythrocyte membrane composition and fluidity in overweight and moderately obese women. J. Nutr. Biochem..

[34] Sinn N., Milte C.M., Street S.J., Buckley J.D., Coates A.M., Petkov J., Howe P.R.C. (2012). Effects of n-3 fatty acids, EPA v. DHA, on depressive symptoms, quality of life, memory and executive function in older adults with mild cognitive impairment: a 6 month randomised controlled trial. Br. J. Nutr..

